# Understanding Intimate Partner Violence Service Delivery for Latinx Survivors in Rural Areas

**DOI:** 10.1177/10778012221140136

**Published:** 2022-12-13

**Authors:** Jeongsuk Kim, Cynthia Fraga Rizo, Christopher J. Wretman, Carolina Alzuru, Deena Fulton, Lisi Martinez Lotz, Brittney R. Chesworth, Ashley D. Givens, Rebecca J. Macy

**Affiliations:** 1 School of Social Work, University of South Florida, Sarasota, FL, USA; 2 School of Social Work, 2331 University of North Carolina at Chapel Hill, Chapel Hill, NC, USA; 3 The Cecil G. Sheps Center for Health Services Research, 2331 University of North Carolina at Chapel Hill, Chapel Hill, NC, USA; 4 North Carolina Counts Coalition, Raleigh, NC, USA; 5 North Carolina Department of Health and Human Services, Raleigh, NC, USA; 6 North Carolina Area Health Education Centers, Chapel Hill, NC, USA; 7 School of Health Professions, University of Missouri, Columbia, MO, USA

**Keywords:** intimate partner violence, Latinx survivors, rurality, service provision, barriers

## Abstract

Using a statewide survey, this exploratory, cross-sectional study examined 78 domestic violence (DV) service organizations’ service delivery practices and perceived challenges to serving Latinx survivors in the context of rurality. Findings showed that DV organizations in rural areas perceived more challenges to delivering culturally appropriate services for Latinx survivors compared to those in other geographic settings even after accounting for client characteristics, service provision characteristics, and community resources. The study finding offers critical insights to ensure and enhance the provision of linguistically and culturally accessible services for rural Latinx survivors of intimate partner violence.

## Background

Intimate partner violence (IPV) is a serious and prevalent problem in the United States (U.S.) among *Latinx* populations that are primarily Spanish-speaking and of Latin American origins. A recent systematic review on the prevalence of interpersonal violence among Latinx women found IPV prevalence rates ranging from 4% to 80%, with about 41% of the studies reporting higher prevalence rates for Latinx women than the U.S. national average (Gonzalez et al., 2020). Along with potentially higher prevalence, Latinx IPV survivors may also experience disproportionately high rates of physical health concerns (e.g., persistent health problems), mental health concerns (e.g., anxiety, depression, posttraumatic stress disorder), and IPV-related homicides compared to their non-Latinx counterparts (Cuevas et al., 2010; Sabina & Swatt, 2015; Stansfield et al., 2019).

Domestic violence (DV) service organizations across the U.S. play an important role in addressing the various needs of all IPV survivors—including Latinx survivors—through services such as advocacy, case management, crisis services, counseling, and shelter (Kulkarni, 2019; Macy et al., 2010, 2018). While this paper uses the phrase IPV, given its broad inclusiveness of all types of violence that can occur among diverse intimate partners, the term DV is used to describe the service organizations because that is their typical title in many U.S. communities. DV service organizations are also increasingly focused on ensuring the provision of culturally appropriate services (Wretman et al., 2021). Despite the availability of such organizations and trends, Latinx survivors may be less likely than other survivors to utilize needed services. Indeed, a national survey found that only 37% of Latinx women who had experienced IPV sought medical help, 27% sought police services, 25% sought legal services, and 14% sought help from social services (Cuevas et al., 2014). Research has found that Latinx survivors’ underutilization of available services may be related to the presence of numerous cultural, socioeconomic, and legal barriers (O’Neal & Beckman, 2017; Rizo & Macy, 2011). Examples of such barriers include antiimmigration beliefs, discrimination, immigration-related fears, limited English proficiency, lack of familiarity with IPV laws and available services, negative prior help-seeking experiences, and social isolation (Alvarez & Fedock, 2018; Lawson et al., 2012; O’Neal & Beckman, 2017; Rizo & Macy, 2011). Notably, all of these barriers are compounded for Latinx survivors living in rural areas (Sawin et al., 2017).

While it is clear that IPV is a serious problem among the large and growing Latinx population in the U.S. and that IPV organizations play a crucial helping role, there is a limited understanding of how such organizations deliver services to Latinx survivors, particularly in rural areas. To better understand IPV service delivery for Latinx survivors living in rural areas, the statewide survey reported herein sought to examine service delivery practices and challenges.

### IPV Service Provision and Rurality

It is well known that rural areas in the U.S. are different than their nonrural (i.e., suburban, urban) counterparts and that this difference often affects peoples’ livelihoods, health, and access to services. Rural settings—being more geographically dispersed and less densely populated—present a unique area of focus for IPV research (Johnson et al., 2014). A systematic review focused on research examining similarities and differences in IPV among rural, urban, and/or suburban areas identified 63 studies (Edwards, 2015). This review found that despite similar prevalence rates of IPV across rural and other areas, IPV perpetrated in rural areas tends to be more chronic and severe, leading to higher rates of IPV-related homicides. Moreover, this review found that survivors in rural areas have worse physical and mental health outcomes compared to those living in nonrural areas and that this disparity is likely because of differences in the availability and accessibility of quality DV services.

In terms of accessibility, research has found that IPV survivors in rural areas face several barriers to accessing services related to their geographic and social isolation (Edwards, 2015; Peek-Asa et al., 2011). For example, rural survivors may face challenges including confidentiality concerns, cultural norms, longer distances to service organizations, and lack of transportation (Eastman & Bunch, 2007; Edwards, 2015; Peek-Asa et al., 2011; Yun et al., 2009; Zielewski & Macomber, 2008).

Related to availability, research has documented that rural areas have fewer dedicated DV organizations and services than urban and suburban areas (Edwards, 2015; Iyengar & Sabik, 2009; Peek-Asa et al., 2011; Yun et al., 2009; Zielewski & Macomber, 2008). Moreover, when available, DV organizations in rural areas tend to experience various organizational barriers to providing quality IPV services (Edwards, 2015). Such organizational barriers to service provision may be related to limited funding and community resources (Gillespie et al., 2019; Yun et al., 2009). Research suggests that DV organizations in rural areas, compared to those in other areas, receive less funding and grants to support their outreach, programming, and service provision (Edwards, 2015; Gillespie et al., 2019; Iyengar & Sabik, 2009; Yun et al., 2009). Adding to these funding issues, limited community resources in rural areas pose additional challenges to DV organizations’ service provision (Johnson et al., 2014). Generally, rural areas have fewer community housing programs, physical and mental health resources, and substance abuse treatment programs. These larger community characteristics affect rural DV organizations’ ability to make timely and appropriate referrals to other community organizations (Johnson et al., 2014; Yun et al., 2009). Given challenges associated with the funding and community resources, staff working in rural DV organizations are generally less prepared and report the need for more training (Eastman & Bunch, 2007; Yun et al., 2009)

### Service Provision for Latinx Survivors in Rural Areas

Frequently, Latinx populations reside in rural and semirural settings in the U.S. Given the unique characteristics and needs of Latinx survivors and rural DV organizations, examining the intersection of service provision for Latinx survivors in rural areas warrants further exploration. Yet, few studies have explored service provision for Latinx IPV survivors with an explicit focus on rurality (Sawin et al., 2017). Nonetheless, there is increasing awareness of the importance of providing DV services that are both linguistically and culturally appropriate (Alvarez & Fedock, 2018; Wretman et al., 2021). Best practices for serving Latinx survivors identified in the literature include engaging in culturally specific outreach, ensuring services and materials are available in Spanish, and hiring bilingual and bicultural staff (O’Neal & Beckman, 2017; Serrata et al., 2020). Research suggests that IPV organizations in rural areas may face organizational barriers to providing linguistically and culturally appropriate services for Latinx survivors (Johnson et al., 2014). For example, rural organizations tend to have fewer bilingual staff, fewer interpreters, and lack diverse service programs (Eastman & Bunch, 2007; Yun et al., 2009). Moreover, given findings related to the training needs of rural service providers, it is likely that such providers lack training on cultural competency and the unique needs of Latinx survivors. For all these reasons, research focused on the landscape of DV organizations and their service provision for Latinx survivors in rural areas is needed.

### Current Study

Overall, despite growing research on IPV in rural areas (Edwards, 2015) and the service needs and barriers of Latinx survivors broadly (O’Neal & Beckman, 2017; Rizo & Macy, 2011), there remains a limited understanding of how DV organizations deliver services to Latinx survivors in a context of rurality. Accordingly, the goal of the present exploratory study is to contribute to this understudied area by examining DV service delivery practices and the perceived challenges to serving Latinx survivors in the context of rurality. Based on the evidence reviewed above, the study outlines four research questions (RQs) to be examined:

RQ #1: What are the service provision characteristics of the DV organizations, especially those relevant to serving Latinx IPV survivors, and do these characteristics vary between rural-only settings and other settings?

RQ #2: What are the available community services for Latinx residents served by the DV organizations, and do these services vary between rural-only settings and other settings?

RQ #3: What are the DV organizations’ reported barriers to serving Latinx IPV survivors, and do these barriers vary between rural-only settings and other settings?

RQ #4: Do the DV organizations in rural-only settings report higher numbers of service delivery barriers after accounting for service provision characteristics and community services?

Thus, the research sought to determine whether a rural setting might be associated with differences in on-the-ground service provision for Latinx IPV survivors and, if so, whether at a more macro-level that rural setting might also be associated with the availability of community services and perceived barriers. Furthermore, this study also sought to explore whether being in a rural setting would be associated with having more perceived barriers even after accounting for service provision characteristics and available community services.

## Methods

The focus of the study was on North Carolina—a state with continuing levels of significant rurality despite recent population growth making it the [rank masked] most populous state in the U.S. For example, recent census data showed that in 64 out of 100 [state masked] counties ≥50% of the population lived in rural areas and in more than one-third of the state's counties (*n* = 36) ≥75% of individuals lived in rural areas (US Census Bureau, 2017). Latinx residents are estimated to number over 1,000,000 in [state masked], comprising approximately 10% of its population and growing. Significant numbers of Latinx [state masked] residents live and work in rural areas in industries related to agriculture, construction, and services.

The study team was a collaboration between members of the North Carolina Coalition Against Domestic Violence (NCCADV)—the state's leading IPV advocacy organization—and researchers from the University of North Carolina at Chapel Hill (UNC-Chapel Hill). The study's overarching aim was to better understand IPV service delivery for Latinx survivors across the state. As such, to the best of the team's knowledge, this effort represented a first-of-its-kind exploration of this issue within North Carolina. The primary activity of the study was a statewide survey of both (a) traditional and (b) culturally specific organizations offering Latinx DV services across all areas of [state masked]. The purpose of the study was to better understand Latinx DV service provision within the state to inform future research and policy, and to broadly inform Latinx DV service provision in the U.S. All research procedures were approved by, and conducted under the auspices of, the Office of Human Subjects Research at UNC-Chapel Hill (15-0503).

### Participants and Procedures

#### Sample

The target population for this investigation consisted of nonprofit human services organizations offering any form of direct DV services in [state masked]. The sampling frame was developed using (a) the official membership list of [organization masked] and (b) targeted online searches. A working database was created by [university masked] team of all identified organizations that included data related to (a) the organization (e.g., name, mailing address, website) and (b) their primary contact (e.g., executive director name, executive director telephone number, executive director email address). All information in the database was verified and checked prior to survey administration. The final sampling frame included 99 separate organizations. Thus, the study employed a cross-sectional design to gather data on a representative sample of the population of [state masked] DV service-providing organizations.

#### Recruitment and survey administration

Recruitment and survey administration occurred concurrently between August 2015 and January 2016. All organization contacts (i.e., executive directors) received a personalized email as well as a personalized mail letter with information about the study, the survey, and an invitation to participate. To encourage participation and reduce respondent burden, participants were given options to complete the survey electronically, by a mailed paper copy, or by telephone. The executive directors, at their discretion, were invited to either complete the survey themselves or to designate a staff member knowledgeable about the organization's Latinx service delivery to complete it instead. Throughout the recruitment and survey administration period, participants were sent reminder emails at scheduled time points. Also, near the end of the period, the research team made select reminder phone calls to organization contacts to further encourage participation and to answer any questions about the study or survey. In appreciation for their time, all participants who completed the survey were eligible for one of three $100 donations to their organization or one of two free registrations for [organization masked]'s annual conference the upcoming year.

### Survey Instrument

The survey instrument itself was developed iteratively over several steps by both parts of the research team (i.e., [organization masked], [university masked]). An initial draft of the survey was created based on (a) extant reviews of research literature on DV Latinx service delivery by [university masked] team members and (b) the expertise of [organization masked] team members. This initial survey was then collaboratively reviewed by all members of the research team to discuss wording, appropriateness, and the inclusion of items. The entire research team then revised the survey. Next, substantive experts in the field (*n* = 3) were invited to review the survey and provide additional feedback on the relevance and appropriateness of the survey items. Lastly, after further revision based on this expert feedback, the final survey was converted to a web-based form using Qualtrics. The web-based version was pilot tested by [organization masked] staff members (*n* = 5) to ensure the survey sequencing worked properly. This pilot testing feedback was then used to finalize the web-based version of the survey.

The final survey comprised over 250 total open- and closed-ended items. Broadly, the survey included items focused on five main content areas: (a) participant characteristics (e.g., demographics, experience); (b) organization characteristics (e.g., location, type, staffing, clients); (c) organization service provision (e.g., available services, practices, outreach); (d) organization attitudes and perceptions (e.g., cultural competence, knowledge, perceived barriers); and (e) community characteristics (e.g., available services, Latinx attitudes).

### Measures

#### Independent variable

For the current analysis, the primary focus was on the independent variable of the *setting*. Based on a single self-report item asking whether the organization “serve[s] a rural, suburban, or urban area,” observations were coded as either “Rural only” or “Others”—a composite category encompassing six different permutations of the three original “Urban” and/or “Suburban” and/or “Rural” options. It was generally assumed that this setting-based variable was also a proxy indicator for the physical location of the organization. Thus, this variable dichotomized organizations into (a) those which only operated in rural areas in [state masked] and (b) those which operated in any other area combination (e.g., “Rural” AND “Suburban”). It should be noted that survey responses were anonymized meaning the data could not be linked to the addresses collected for recruitment purposes, thus precluding a more exact determination of the organizations’ setting.

#### Dependent variables

The analyses featured three dependent variables for (a) *Latinx IPV service provision*, (b) *Latinx community services*, and (c) *Latinx IPV service barriers*.

Latinx IPV service provision included 15 variables across four domains: (a) staff characteristics (*n* = 6; e.g., “# of full-time staff members”), (b) training characteristics (*n* = 2; e.g., “% of staff attending cultural competency training”), (c) language utilization characteristics (*n* = 4; e.g., “% of IPV materials in Spanish”), and (d) Latinx outreach characteristics (*n* = 3; e.g., “Has a medium-to-high presence in their Latinx community”). Twelve of these variables were single items. The remaining variables were multiitem total composites: (a) “# of direct services provided in Spanish” (0–17), “# of strategies used to address language needs of Latinx clients” (0–8), and “# of outreach activities conducted in Latinx community related to IPV” (0–14). Overall, these 15 service provision variables covered both general DV service provision and Latinx-specific service provision. All variables were coded such that a greater number, high percentage, or “Yes” answer was indicative of more culturally appropriate DV service provision for Latinx survivors.

Latinx community services included 11 delineated community services selected by the research team plus a composite “Total” variable (0–11) for the organization-level total count. Participants checked each individual service item if it was “available for Latinas/os in the community served by [their organization]” to “the best of [their] knowledge.” The 11 services were “culturally appropriate mental health services,” “legal aid,” “immigration services,” “financial/economic assistance,” “ESL or ESOL classes” (i.e., English as a second language or English to speakers of other languages), “culturally appropriate employment assistance,” “homeless shelter,” “transitional housing,” “batterers programs/services,” “culturally appropriate DV services,” and “DV shelter.”

Latinx DV service barriers included 26 delineated perceived barriers to providing culturally appropriate services to Latinx IPV survivors identified by the research team plus a composite “Total” variable (0–26). Participants responded using a 5-point scale ranging from “Strongly disagree” to “Strongly agree” to all 26 items which included perceived barriers related to the availability of childcare, DV services, employment assistance, mental health agencies, shelter services, substance abuse agencies, transitional housing; availability of Latinx service staff, on-site interpreters, phone-in interpretation services, availability of Spanish-speaking service staff; cultural disconnect; documentation status; funding; knowledge about DV; knowledge about Latinx culture and needs; newness of migration destination; policy; remoteness of agency location; remoteness of service area; size of agency; size of service area; stigma; training; transiency; and transportation. Thus, the items comprised a mix of (a) items directly related to IPV (e.g., “Availability of appropriate DV shelter services for Latina/o clients”) and (b) generalized items related to services for Latinx people (e.g., “Training about culturally relevant best-practices”). For analytical purposes, the response options of “Strongly agree” and “Agree” were collapsed into “Agree/Yes” (1) and all others into “Disagree/No” (0).

### Data Analysis

All analytic tasks were conducted using Stata 16.1 (StataCorp, College Station, TX) from 2020 to 2021. A prespecified statistical significance level of *p* < .05 (two-sided) was used throughout.

#### Data cleaning

Prior to outcome analyses, data were thoroughly checked for missingness, out-of-range errors, and interitem incongruence errors. Observations were also checked for (a) abnormally fast completion times and (b) high levels of participant-level missingness. Also, observations were checked to determine whether participants had completed key variables related to (a) setting and (b) recent provision of services to survivors.

#### Descriptive analyses

Descriptive analyses involved two major steps designed to better understand IPV service provision among the sample of organizations. First, the three sets of dependent variables were summarized using univariate statistics (e.g., mean [*M*], standard deviation [*SD*], frequency [*n*], proportion [%]) to describe the nature of the analytic sample. Second, bivariate analyses summarized descriptive statistics by setting and testing for a significant difference using (a) Wilcoxon rank-sum tests for continuous variables and (b) Fisher's exact tests for nominal variables. These tests sought to determine where differences existed in organizations’ Latinx DV service provision, community services, and perceived DV service barriers in rural versus other settings.

#### Multivariable models

The final analytic step was to test multivariable models that would add context to whether a rural setting is associated with having more of the 26 delineated barriers. Note that this step was undertaken out of a desire to account for important characteristics (i.e., service provision, community resources) that might confound the relationship between rurality and the barriers in the analytic sample herein, rather than to model generalizable population-level estimates. Preliminary checks revealed that 14 of 74 (18.9%) participating organizations that completed any of the barrier items did not complete all 26 of them. Accordingly, the multivariable analysis needed to account for the fact that the denominator (i.e., # of barriers questions answered) varied across organizations and model the barriers dependent variable not as a count outcome but as a continuous proportion.

Thus, an overarching multivariable modeling approach was chosen that used a binomial logistic regression with a logit link appropriate for the grouped data (i.e., barriers aggregated to organization-level) where the dependent variable was a numerator representing the number of “Agree/Yes” responses and a population variable was included that was the total number of barriers responses (i.e., the denominator of ≤26 counts of barrier item responses). This approach estimated results via maximum likelihood using Stata's default version of the Newton–Raphson algorithm. Further, standard errors (SEs) were adjusted for potential misspecification as recommended with the robust Huber/White sandwich estimator (Huber, 1967; White, 1980). Specification of the logit link was tested using a post hoc goodness-of-link test with matching binomial distribution and robust error specifications whereby the dependent variable was regressed on the predicted value and its square such that a nonsignificant value for the square of the predicted value being an indication of the appropriateness of the link and, thus, a correctly specified model. Model-estimated individual parameters were reported as coefficients exponentiated to produce adjusted odds ratios (AORs) and their corresponding SEs and 95% confidence intervals (CIs). Under this framework, an AOR of >1.00 for the setting independent variable was thus indicative of greater barriers faced. Overall model characteristics were reported including analytic sample size (*n*), degrees of freedom (df), log pseudolikelihood, and the Akaike information criterion (AIC). The AIC was included specifically to compare fit across models (see below) and was a version that adjusts for the sample size as −2LL + 2*k*/*n*, where LL is the model log pseudolikelihood produced due to the robust variance–covariance matrix, *k* is the number of model parameters, and *n* is the number of observations. Models with lower AIC values (i.e., closer to zero) represent those with the better fit (Akaike, 1998).

The multivariable analytic strategy outlined a priori a multimodel approach analogous to a sensitivity analysis of the conceptual relationship between rurality and barriers. Three model forms were tested. First, Model #1 featured the rurality independent variable plus three organization covariates intended to account for core differences in clients served: (a) # of total clients served, (b) % of clients that were Latinx, and (c) % of clients that spoke primarily Spanish. Model #2 extended this model by adding key Latinx service provision characteristics. To emphasize parsimony, the total of 15 characteristics were reduced via backward stepwise selection until only those with a *p* < .05 significance level remained. Model #3 represented a further extension that added the total count of available Latinx community services (0–11). Note that in all three models due to some high values for the # of total clients served covariate, a correction was undertaken whereby values were classified as outliers if they were >2.5 the median absolute deviation as recommended by experts (Leys et al., 2013). These outliers were then winsorized to the most proximal highest value. No other independent variables or covariates were transformed in any way. Missing values on variables resulted in the listwise deletion of observations.

## Results

### Organizations

Of 99 organizations recruited, 82 (82.8%) participated in the survey. Among those, two exclusion criteria removed those that either (a) reported not serving any clients in the previous year (*n* = 2) or (b) did not answer the item indicating their setting (*n* = 2). Diagnostic checks determined that no observations should be excluded due to completion time or participant-level proportion of missingness. The final analytic sample was thus all 78 who served actual clients in the previous year (i.e., 2014) regardless of setting, characteristics, responses, or missing data.

Consistent with many [state masked] counties having ≥50% of their residents in rural areas, the majority of organizations were “Rural only” (*n* = 50, 64.1%) compared to the remaining organizations (*n* = 28, 35.9%) that operated in some mixture of urban, suburban, and rural settings.

### Latinx IPV Service Provision

Table 1 shows the 15 IPV service provision characteristics across the four delineated domains. Overall, the surveyed organizations were considered to be likely representative of the population of those operating within [state masked]. The mean number of full-time staff was 9.65 with a majority (58.1%) of organizations reporting having ≥1 Latinx and/or Spanish-speaking full-time staff member. Training levels were moderate with a mean of 54.30% of staff having undergone cultural competency training and 40.00% Latinx services training. A mean of 7.68 of 17 possible direct services was provided in Spanish and on average a majority (58.11%) of IPV-specific materials were in Spanish. Latinx outreach was measurable with a mean of 5.76 of 14 outreach activities conducted in the Latinx community and 64.4% of organizations reported a moderate-to-strong relationship with their Latinx community. Not pictured, though important contextually, is that aside from one outlier organization which reported serving almost 10,000 clients, the organizations reported serving a mean of 754.10 clients in the previous year (median = 450.00) of whom a mean of 26.64% was Latinx and a mean of 24.72% spoke primarily Spanish.

**Table 1.** Domestic Violence (DV) Organizations’ Latinx Service Provision Characteristics, by Setting (*N* = 78).

DV organizations’ Latinx service provision characteristics	Setting *M* (*SD*) or *n* (%)			*p*
	All (*n* = 78)	Rural only (*n* = 50)	Others^a^ (*n* = 28)	
Staff				
# of full-time staff members	9.65 (9.73)	7.08 (5.16)	14.25 (13.69)	.021
Has ≥1 Latinx and/or Spanish-speaking full-time staff member	43 (58.1)	22 (46.8)	21 (77.8)	.014
# of part-time staff members	6.39 (7.06)	5.10 (4.74)	8.74 (9.67)	.23
Has ≥1 Latinx and/or Spanish-speaking part-time staff member	29 (40.3)	14 (29.8)	15 (60.0)	.022
# of volunteers	23.48 (34.42)	16.18 (23.52)	37.23 (46.31)	.030
Has ≥1 Latinx and/or Spanish-speaking volunteer	48 (66.7)	30 (63.8)	18 (72.0)	.60
Training				
% of staff attending cultural competency training	54.30 (33.79)	50.85 (34.05)	61.04 (32.94)	.23
% of staff attending Latinx services training	40.00 (32.85)	38.82 (32.69)	42.12 (33.70)	.71
Language utilization				
# of direct services provided in Spanish (0–17)	7.68 (4.36)	7.46 (4.45)	8.07 (4.25)	.46
# of strategies used to address language needs of Latinx clients (0–8)	2.81 (1.82)	2.66 (1.92)	3.07 (1.61)	.23
% of IPV materials in Spanish	58.11 (32.11)	53.98 (32.60)	65.12 (30.61)	.15
Has some website materials available in Spanish	26 (36.1)	11 (23.9)	15 (57.7)	.006
Latinx outreach				
# of outreach activities conducted in Latinx community related to IPV (0–14)	5.76 (3.35)	5.40 (3.27)	6.39 (3.46)	.16
Has a medium-to-high presence in their Latinx community	39 (52.0)	21 (44.7)	18 (64.3)	.15
Has a moderate-to-strong relationship with their Latinx community	47 (64.4)	28 (60.9)	19 (70.4)	.46

*Note*. IPV = intimate partner violence; *SD* = standard deviation. Due to missing data, response frequencies range from 70 to 78 organizations among variables. *p*-value compares “Rural only” versus “Others” organizations using either a two-sided Wilcoxon rank-sum exact test (continuous) or Fisher's exact test (nominal). Data collected via organization director self-reports from August 2015 through January 2016 in [state masked]; All responses related to 2014 service provision.

^a^ Includes six permutations of rural and/or suburban and/or urban settings.

When comparing organizations by setting, several significant differences were noted. When compared with their counterparts, those 50 organizations serving rural-only locations were found to (a) have fewer full-time staff members (−7.2 members; *p* = .021), (b) be less likely to have Latinx and/or Spanish-speaking full-time staff (−31.0%; *p* = .014), (c) be less likely to have Latinx and/or Spanish-speaking part-time staff (−30.2%; *p* = .022), (d) have fewer volunteers (−21.1 volunteers; *p* = .030), and (e) have fewer online materials in Spanish (−33.8%; *p* = .006).

### Latinx Community Resources

Table 2 shows perceptions of available resources for Latinx survivors in their communities with only those resources having a >25.0% availability for Latinx survivors presented for parsimony. Of the 11 total services, organizations reported a mean of 4.19 of these resources as being available. The three most common individual services were (a) ESL/ESOL classes (79.5% of organizations), (b) legal aid services (69.2%), and (c) financial/economic assistance (47.4%). Availability of IPV-specific services was low with only 16.7% (*n* = 13) of organizations reporting having transitional housing and 9.0% (*n* = 7) having a shelter for IPV survivors (not pictured).

**Table 2.** Domestic Violence Organization-Reported Latinx Community Services, by Setting (*N* = 78).

Latinx community services	Setting *M* (*SD*) or *n* (%)			*p*
	All	Rural only (*n* = 50)	Others^a^ (*n* = 28)	
Total available community services (0–11)^b^	4.19 (2.28)	3.74 (2.16)	5.00 (2.31)	.010
ESL/ESOL classes	62 (79.5)	39 (78.0)	23 (82.1)	.78
Legal aid	54 (69.2)	30 (60.0)	24 (85.7)	.022
Financial/economic assistance	37 (47.4)	24 (48.0)	13 (46.4)	1.00
Immigration services	35 (44.9)	17 (34.0)	18 (64.3)	.017
Culturally appropriate mental health services	31 (39.7)	16 (32.0)	15 (53.6)	.091
Batterers' programs/services	29 (37.2)	11 (22.0)	18 (64.3)	<.001
Homeless shelters	29 (37.2)	18 (36.0)	11 (39.3)	.81
Culturally appropriate employment assistance	23 (29.5)	17 (34.0)	6 (21.4)	.31

*Note*. *SD* = standard deviation; ESL = English as a second language; ESOL = English to speakers of other languages. There are no missing data among variables. *p*-value compares “Rural only” versus “Others” organizations using either a two-sided Wilcoxon rank-sum exact test (continuous) or Fisher's exact test (nominal). Data collected via organization director self-reports from August 2015 through January 2016 in [state masked]. Only services with ≥25.0% prevalence among all organizations presented (transitional housing service, domestic violence shelter, and culturally appropriate domestic violence services were analyzed but not presented); Services ordered by frequency from highest to smallest.

^a^ Includes six permutations of rural and/or suburban and/or urban settings.

^b^ Includes all 11 different delineated community services.

Once again, organizations serving rural only areas were often statistically and meaningfully different than their counterparts, being found to (a) have fewer overall services (−30.2%; *p* = .022), (b) be less likely to have legal aid (−30.2%; *p* = .022), (c) be less likely to have immigration services (−30.2%; *p* = .022), and (d) be less likely to have batterers programs or services (−30.2%; *p* = .022) among the eight resources presented and tested in Table 2. Overall, nine of 11 services were less likely to be found in rural only areas.

### Latinx DV Service Barriers

Table 3 shows providers’ perceptions of barriers related to their IPV service provision for Latinx survivors, with only those barriers having a ≥50.0% prevalence presented. Four organizations did not complete any of these items for reasons unknown. Of the 26 total barriers reported by the other 74 organizations, a large number were noted with an overall mean of 13.03 barriers (*SD* = 5.70). Over one-third (36.5%, *n* = 27) of organizations reported ≥15 barriers. The three most noted barriers were the availability of childcare (83.3% of organizations), transportation limitations (77.8%), and funding (71.8%). The three least commonly reported barriers (not pictured) were (a) federal and/or state policies (30.0%), (b) knowledge about IPV (25.7%), and (c) being in an area that is a new destination for Latinx immigrants (23.6%).

**Table 3.** Domestic Violence Organization-Reported Latinx IPV Service Barriers, by Setting (*N* = 74).

Latinx IPV service barriers	Setting *M* (*SD*) or *n* (%)			*p*
	All	Rural only (*n* = 47)	Others^a^ (*n* = 27)	
Total barriers to serving Latinx IPV survivors (0–26)^b^	13.03 (5.70)	14.40 (5.84)	10.63 (4.65)	.007
Availability of childcare	60 (83.3)	36 (82.2)	23 (85.2)	1.00
Transportation limitations of Latinx clients	56 (77.8)	37 (82.2)	19 (70.4)	.26
Funding	51 (71.8)	34 (73.9)	17 (68.0)	.59
Availability of transitional housing	50 (67.6)	29 (61.7)	21 (77.8)	.20
Availability of employment assistance	49 (67.1)	28 (60.9)	21 (77.8)	.20
Availability of mental health agencies	47 (64.4)	30 (63.8)	17 (65.4)	1.00
Availability of shelter services	46 (63.0)	30 (65.2)	16 (59.3)	.63
Availability of substance use agencies	45 (60.8)	27 (57.5)	18 (66.7)	.47
Availability of Latinx service staff	44 (59.5)	35 (74.5)	9 (33.3)	.001
Latinx stigma regarding seeking services	43 (58.9)	29 (63.0)	14 (51.9)	.46
Availability of Spanish-speaking service staff	43 (58.1)	31 (66.0)	12 (44.4)	.089
Availability of on-site interpreters	42 (56.8)	33 (70.2)	9 (33.3)	.003
Remoteness of the area served by the organization	36 (50.7)	30 (66.7)	6 (23.1)	.001

*Note*. IPV = intimate partner violence; *SD* = standard deviation. Analyses completed on the 74 organizations out of the total of 78 (94.9%) that completed ≥1 of the 26 barriers items in the survey. Due to missing data, response frequencies range from 71 to 74 organizations among variables The presence of reported barriers noted by respondents as either “Agree” or “Strongly agree” versus “Neither agree nor disagree,” “Disagree,” or “Strongly disagree.” *p*-value compares “Rural only” versus “Others” organizations using either a two-sided Wilcoxon rank-sum exact test (continuous) or Fisher's exact test (nominal). Data collected via organization director self-reports from August 2015 through January 2016 in [state masked]. Only barriers with ≥50.0% prevalence of “Agree” or “Strongly agree” among all organizations presented (availability of phone-in interpretation services, knowledge about Latinx culture and needs, knowledge about domestic violence, documentation (i.e., legal status) of Latinx immigrants, availability of domestic violence shelter services for Latinx referral, availability of appropriate domestic violence agency for Latinx referral, size of the service area, size of agency, training about culturally relevant best practices, newness of area as a migration destination for Latinx immigrants, transiency of the Latinx population, cultural disconnect with Latinx community, federal or state policies were analyzed but not presented); Barriers ordered by frequency from highest to smallest.

^a^ Includes six permutations of rural and/or suburban and/or urban settings.

^b^ Includes all 26 different delineated barriers.

Barriers were frequently more commonly reported by organizations in rural only areas. Whereas rural-only organizations reported facing a mean of 14.40 barriers, organizations in other areas perceived experiencing only 10.63 barriers (−26.2% relative decrease; *p* = .007). Other significantly more common barriers for rural-only organizations were (a) availability of Latinx service staff (i.e., less availability; +41.2% of organizations; *p* = .001), (b) availability of on-site interpreters (i.e., less availability; +36.9%; *p* = .003), and (c) remoteness of the area served by the organization (i.e., more remote; +43.6%; *p* = .001) among the 13 barriers presented and tested in Table 3.

### Multivariable Models

Data were first checked for missing values prior to testing the models. In total, 60 (81.1%) of the 74 organizations that completed any barriers items were found to have completed all 26. Among those 14 organizations that did not, nine organizations did not complete one item, two did not complete two items, one each did not complete three and five items, and one organization did not complete 15. Wilcoxon rank-sum exact and Fisher's exact tests comparing full completers versus others did not find statistically significant differences between the two groups of observations based on the service provision characteristics (All: *p* ≥ .064; 13 of 15: *p* ≥ .21). Among the 26 barriers items, seven had zero missing values and none had more than five missing values. Missingness was assumed to be completely at random.

After aggregating data at the organization level, the barriers proportion dependent variable had a mean of 0.514 (i.e., 51.4% of possible barriers reported). The top quartile (i.e., ≥75th percentile) of organizations (*n* = 20, 27.0%) reported having a barriers proportion of ≥0.654.

Table 4 presents the results of the three multivariable models. Model #1 including the three organization covariates found that being in a rural-only setting was significantly associated with greater odds of reporting barriers (AOR = 1.81, *SE* = 0.44; 95% CI: 1.13, 2.90; *p* = .014). The model analytic sample size was *n* = 69 due to missing data and the AIC was 8.68. The goodness-of-link test was nonsignificant indicating likely correct specification of the model using the logit link (*p* = .42). Nevertheless, a sensitivity analysis testing alternative models using (a) probit, (b) log–log, and (c) complementary log–log links (not pictured) was conducted which resulted in similar individual parameter estimates (rural-only setting AOR: 1.45, 1.53, and 1.54, respectively) and almost identical model fit (AIC = 8.67, 8.69, and 8.66, respectively).

**Table 4.** Multivariable Models of Domestic Violence Organization-Reported Proportion of Latinx Intimate Partner Violence Service Barriers on Rurality, Organization Covariates, and Key Independent Variables (*n* = 74).

Variables and model characteristics	Model #1 AOR (*SE*); 95% CI (*p*)	Model #2 AOR (*SE*); 95% CI (*p*)	Model #3 AOR (*SE*); 95% CI (*p*)
Rural only setting	1.81 (0.44); 1.13, 2.90 (.014)	3.37 (0.88); 2.03, 5.61 (<.001)	3.72 (1.07); 2.11, 6.55 (<.001)
Organization covariates			
# of total clients served (unit = 100)	0.99 (0.03); 0.92, 1.06 (.75)	0.98 (0.04); 0.91, 1.06 (.64)	0.99 (0.04); 0.92, 1.06 (0.78)
% of clients that were Latinx (unit = 10%)	0.89 (0.05); 0.80, 1.00 (.043)	1.01 (0.05); 0.91, 1.12 (.89)	0.99 (0.06); 0.89, 1.11 (0.89)
% of clients that spoke primarily Spanish (unit = 10%)	1.15 (0.07); 1.02, 1.28 (.019)	1.14 (0.06); 1.03, 1.25 (.011)	1.15 (0.06); 1.03, 1.28 (.010)
Independent variables			
Latinx service characteristics^a^			
# of volunteers (unit = 5)		1.03 (0.01); 1.01, 1.05 (.011)	1.03 (0.01); 1.01, 1.05 (.007)
% of staff attending cultural competency training (unit = 10%)		1.14 (0.06); 1.02, 1.27 (.026)	1.12 (0.06); 1.01, 1.25 (.038)
% of staff attending Latinx services training (unit = 10%)		0.87 (0.05); 0.77, 0.98 (.023)	0.87 (0.05); 0.78, 0.98 (.020)
Has a moderate-to-strong relationship with their Latinx community		0.54 (0.13); 0.34, 0.85 (.008)	0.55 (0.12); 0.35, 0.85 (.007)
Community services for Latinx residents (0–11)			1.06 (0.06); 0.95, 1.18 (.28)
Model characteristics			
*N*	69	57	57
*df*	64	48	47
Log pseudolikelihood	−294.38	−203.93	−201.53
AIC	8.68	7.47	7.42
Goodness-of-link test *p*	.42	.78	.96

*Note*. AOR = adjusted odds ratio; *SE* = robust standard error; CI = confidence interval; df = degrees of freedom; AIC = sample size adjusted Akaike information criterion. The analytic sample comprises the 74 organizations out of the total of 80 (92.5%) that completed the setting item and ≥1 of the 26 barrier items in the survey. Model = binomial logistic regression where the dependent variable was a numerator representing the number of “Agree/Yes” responses and a population variable was included that was the total number of barriers responses, logit link, binomial distribution, maximum likelihood estimation, and robust standard errors.

^a^ Truncated set of covariates selected from an initial pool of 15 via backward stepwise selection so that for all: *p* < .05.

For Model #2, the backward stepwise selection process determined that just four of the 15 Latinx service provision characteristics should be included: (a) # of volunteers, (b) % of staff attending cultural competency training, (c) % of staff attending Latinx services training, and (d) has a moderate-to-strong relationship with their Latinx community. The Model #2 (*n* = 57) estimate for the rurality independent variable remained significant though stronger with an AOR of 3.37 (*SE* = 0.88; 95% CI: 2.03, 5.61; *p* < .001). The Model #2 goodness-of-link test was .78 and the AIC was 7.47—indicative of a moderately better fit than Model #1. Not pictured, a sensitivity analysis testing Model #2 with all 15 Latinx service provision characteristics resulted in a reduced analytic sample size (*n* = 45) but nevertheless found rurality to remain significant (*n* = 45; AOR = 4.38; 95% CI: 2.07, 9.30; *p* < .001).

Finally, the Model #3 estimate for rurality was once again significant: AOR of 3.72 (*SE* = 0.88); 95% CI: 2.11, 6.55; *p* < .001). The Model #2 AIC was 7.47—indicative of a slightly better fit than Model #2. Model #3 was the summative and best model. To elaborate, the final AOR of 3.72 for rurality is interpreted as an estimate that for IPV service organizations in [state masked], the odds of having more barriers to Latinx service provision are 3.72 times higher for those serving rural-only settings and clients compared with their counterparts. Among organization covariates in Model #3, only % of clients that spoke primarily Spanish was significant (AOR = 1.15; *p* < .010). All four Latinx service characteristics were significant (*p* ≤ .038), but the total count of community services for Latinx residents was not significant (*p* = .28).

Listwise deletion of observations due to missing values on variables resulted in an analytic sample size of just 57 in Model #2 and Model #3. Post hoc tests found that among these organizations the frequency of being in a rural-only setting was 63.2% (*n* = 36) and the of total reported barriers was 13.37 (*SD* = 5.89)—values almost identical to the overall sample values of 64.1% and 13.03, respectively. Bivariate tests comparing those included in the final model versus those that were deleted demonstrated significant differences only for # of full-time staff members (analytic sample: *M* = 11.09, deleted sample: *M* = 8.17; *p* = .010), and # of outreach activities conducted in Latinx community related to IPV (analytic sample: *M* = 6.69, deleted sample: *M* = 4.97; *p* = .018).

## Discussion

Despite growing research suggesting differences in IPV experiences and DV service provision based on geographic area (Edwards, 2015), there is limited research on the intersection of service provision for Latinx survivors in the context of rurality. This evidence gap is notable given the heightened awareness that DV services should be both linguistically and culturally appropriate (Alvarez & Fedock, 2018; Serrata et al., 2020), as well as research suggesting that organizations in rural areas may face more barriers to providing DV services generally (Edwards, 2015). Thus, to begin to help develop evidence to address this gap, the current study sought to explore DV service delivery practices and perceived challenges to serving Latinx IPV survivors among DV organizations in rural-only settings compared to those in other geographic settings. Although this exploratory study found some similarities between DV organizations in rural only and other areas, this work more consistently found that DV organizations in rural-only settings are different than their counterparts in terms of key service provision characteristics, available community resources, and perceived challenges to delivering culturally appropriate services. Though preliminary in nature, study findings highlight important implications.

### Service Provision Characteristics

Regarding RQ #1, there were some key similarities and differences between DV service organizations in rural only and other settings. In terms of staffing, participants from rural-only DV organizations reported having significantly fewer full-time staff and volunteers, as well as fewer full- and part-time staff that identified as bilingual or bicultural. These findings support prior research noting gaps in staffing, particularly bilingual/bicultural staffing, for rural DV organizations (Edwards, 2015). For example, Eastman & Bunch (2007) found that compared to providers working at urban DV organizations, those working in rural settings were more likely to perceive their organization as being understaffed.

Analyses discovered no significant differences between rural only and other organizations related to staff members’ participation in training about cultural competency and services for Latinx survivors. This finding was surprising considering prior evidence suggesting staff working in rural DV organizations generally receive less training than their counterparts working in other areas (Edwards, 2015). Overall and across locations, on average just about half of the staff had attended cultural competency training and less than half had attended training on Latinx services. Therefore, there seems to be a need for greater staff training on issues related to cultural competency and serving Latinx survivors regardless of geographic location.

A key component of providing culturally appropriate services is ensuring that services are available in a client's preferred language (Alvarez & Fedock, 2018; O’Neal & Beckman, 2017; Postmus et al., 2014). Findings concerning the linguistic appropriateness of services suggest room for growth across both rural only and other DV organizations. Notably, of the 17 services included in the survey, on average participants reported that only about half of those were available in Spanish. Moreover, few organizations had some of their website materials available in Spanish, with participants from rural-only DV organizations being significantly less likely to report such language capacity. Given the study finding that rural DV organizations have fewer Latinx and Spanish-speaking staff, it is possible that these organizations have less capacity for ensuring their website content is available in multiple languages. It is also possible that these organizations employ other strategies for sharing information about IPV and their organization with the Latinx community.

### Available Community Services

Community resources are critical to providing timely and appropriate services and referrals for Latinx IPV survivors. However, related to RQ #2, the study findings suggest that participants perceived relatively few services available for Latinx survivors in their communities. Moreover, participants from rural-only DV organizations were more likely than those from other DV organizations to report that services for Latinx survivors were unavailable in their community. This finding aligns with previous research suggesting that rural communities may have limited resources and supports necessary for addressing the needs of IPV survivors (Gillespie et al., 2019; Yun et al., 2009). Given that Latinx survivors may often have multiple needs related to education, employment, immigration, and transportation (Bosch & Schumm, 2004; Eisenman et al., 2009; Grossman et al., 2005; Sabri et al., 2018), the lack of available community services—particularly in rural areas—may, in turn, limit DV organizations’ ability to connect clients to essential services for enhancing safety and empowerment.

Specific services that were less likely to be available in rural only communities included legal aid, immigration services, and programs and services for those who perpetrate IPV. Immigration and legal assistance are arguably two of the most essential services needed by immigrant Latinx survivors (Eisenman et al., 2009; Sabri et al., 2018). Accordingly, this study's findings suggest that DV service providers’ ability to address the pressing needs of their Latinx clients may be hindered by the limited availability of these services in rural areas. Moreover, given research suggesting that individuals who perpetrate IPV in rural areas tend to inflict violence with greater chronicity and severity compared to those in urban areas (Edwards, 2015), it is concerning that rural communities may have fewer services for those who perpetrate IPV.

### Perceived Barriers

Related to RQ #3, the study identified multiple perceived barriers to providing culturally appropriate services to Latinx IPV survivors. Overall, key barriers reported by participants from rural only and other DV organizations included the availability of childcare, transportation challenges experienced by Latinx clients, and funding. However, the findings suggest that rural-only DV organizations face a greater number of barriers to providing culturally appropriate services to Latinx survivors compared to organizations in other areas. This finding supports prior evidence that rural DV organizations generally experience more organizational barriers to service provision than those in other geographic areas (Eastman & Bunch, 2007; Edwards, 2015; Gillespie et al., 2019; Yun et al., 2009). In this study, the specific barriers more likely to be reported by participants from rural-only DV organizations included the availability of Latinx service staff, the availability of on-site interpreters, and the remoteness of the area served by the organization. These findings are consistent with other study findings related to differences between rural-only and other DV organizations in terms of staffing, language capacity, and the availability of community services.

The study findings concerning RQ #4 further illuminate differences in perceived barriers between rural-only DV organizations and organizations in other areas. Even after accounting for differences in client characteristics, service provision characteristics, and community resources, rural-only DV organizations still face significantly more perceived barriers to appropriately serving Latinx IPV survivors. This finding suggests that additional environmental factors are likely affecting services for Latinx survivors in rural areas. For example, given research suggesting differences in attitudes regarding immigration and immigrants across geographic settings—with more negative attitudes in rural areas (Garcia & Davidson, 2013), it is possible that potential differences in attitudes regarding Latinx and immigrant populations across these settings may be affecting perceived barriers to delivering culturally appropriate services.

### Implications

To enhance the cultural appropriateness of their services and organization, rural DV organizations should consider innovative strategies for recruiting, hiring, and retaining Latinx and Spanish-speaking staff (O’Neal & Beckerman, 2017; Parson et al., 2016; Postmus et al., 2014). In addition to helping ensure the organization could provide services and organizational materials in Spanish, through their knowledge and understanding of the community, such providers could offer expertise concerning nuanced ways of enriching the cultural appropriateness of services by centering cultural identity, practices, and beliefs (Serrata et al., 2020). It has also been recommended that English-speaking individual DV providers learn key phrases in Spanish to ensure that Spanish-speaking Latinx survivors feel welcomed when seeking services (O’Neal & Beckerman, 2016).

Rural DV organizations also need to develop strategies for overcoming challenges related to having fewer available community services. For example, rural DV organizations could develop collaborative partnerships with organizations in neighboring communities or regions by using virtual platforms. Although such approaches might be novel, they could also offer innovation in addressing IPV and meeting the diverse needs of survivors (Neill & Hammatt, 2015). Notably, prior to COVID-19, there had been some hesitation around virtual collaboration for DV services because of confidentiality concerns. However, the increased use of virtual platforms to collaborate with partners and deliver services during the pandemic has demonstrated that such collaborations are possible and therefore could benefit DV organizations in rural areas.

Regardless of geographic area, the study findings also suggest the need for ensuring more DV providers receive training related to cultural competency and services for Latinx survivors. Esperanza United—a national Latinx organization focused on preventing and ending IPV—offers technical assistance and an array of training focused on culture, service provision for Latinx survivors, community engagement and outreach, and building organizational capacity for serving Latinx survivors. Partnering with Esperanza United or other similar organizations may help DV service organizations, especially those in rural only areas, address organizational barriers to providing culturally appropriate services to Latinx survivors. In addition to advocating for the need for training related to cultural competency and services for Latinx survivors within their organizations, DV providers could also independently explore the availability of such training as part of their own personal and professional development.

Policymakers and funders could also consider efforts to enhance the accessibility and availability of quality IPV services for Latinx survivors, particularly in rural areas. Notably, over 70% of organizations identified funding as a perceived barrier to providing culturally appropriate services for Latinx survivors. To address this potential barrier, policymakers and funders could create specific funding streams for the provision of services targeting Latinx survivors. Specific funding mechanisms could also be created for rural DV organizations. In addition to developing specific funding calls to address the needs of rural organizations and Latinx survivors, policymakers and funders could also review funding requirements and allowable costs to ensure that these criteria increase rather than hinder the accessibility and availability of services. For example, study findings suggest that efforts to develop strong relationships with the Latinx community through outreach should be an allowable cost. Moreover, access to on-demand interpretation (e.g., language line) could be added as a requirement for receiving funding.

Although this study begins to expand understanding of service delivery practices and organizational challenges to serving Latinx survivors in the context of rurality, there is a need for more research in this area. Future research should explore the relationship between organizational barriers, cultural competency, and Latinx survivor outcomes, as well as whether the relationships among these variables vary based on rurality. Future research is also needed on rural Latinx survivors’ experiences accessing DV services and their perceptions on the cultural appropriateness of those services.

### Limitations

There are, of course, limitations to this exploratory study. First, it should be clear that the analytic sample is small and limited to the population of DV organizations in [state masked]. The providers and organizations surveyed and studied herein may not be representative of those in other states in the U.S. In addition, all responses are based on organizational contacts’ subjective perceptions as no data were administratively derived nor collected on or from individual survivors. It is possible, therefore, that the responses to items may be reflective of an individual's opinion and may not fully represent their organization's characteristics. Lastly, organizations’ locations have been classified broadly based on participant reports and, unfortunately, could not be more explicitly defined using actual addresses to geocode organizations with rural–urban continuum codes for more accuracy. Accordingly, future research efforts could valuably build from this study to investigate DV service organizations broadly, analyze organizations’ administrative data, collect organizations’ addresses information, and invite Latinx survivors to participate in studies to offer their perspectives on services to researchers directly.

## Conclusion

To the best of our knowledge, this exploratory study is one of the first efforts to investigate service delivery practices and organizational barriers to serving Latinx IPV survivors, and to examine differences based on rurality. The study's findings offer important insights regarding differences across DV organizations in terms of service provision characteristics, available community services, and organizational barriers to providing culturally appropriate services for Latinx survivors. Notably, DV organizations in rural only areas experience more organizational barriers to serving this community than their counterparts in other areas. Although there is a need for more research exploring the intersection of service provision for Latinx survivors in the context of rurality, hopefully, this study offers initial findings on which future efforts can build.
